# L1 cell adhesion molecule (L1CAM) is a strong predictor for locoregional recurrences in cervical cancer

**DOI:** 10.18632/oncotarget.20976

**Published:** 2017-09-18

**Authors:** Marlies Schrevel, Willem E. Corver, Margit E. Vegter, Natalja T. Ter Haar, Enno J. Dreef, Jogchum J. Beltman, Gemma Kenter, Tjalling Bosse, Cornelis D. de Kroon, Ekaterina S. Jordanova

**Affiliations:** ^1^ Department of Pathology, Leiden University Medical Center, Leiden, The Netherlands; ^2^ Department of Gynecology, Leiden University Medical Center, Leiden, The Netherlands; ^3^ Department of Gynecology, VUmc, Centre for Gynecologic Oncology, Amsterdam, The Netherlands

**Keywords:** cervical cancer, epithelial mesenchymal transition (EMT), L1 cell adhesion molecule (L1CAM), locoregional recurrence, prognosis

## Abstract

**Background:**

L1 cell adhesion molecule (L1CAM) has been shown to be a prognostic marker in various cancer types, and has been suggested to play a role in epithelial mesenchymal transition (EMT). Here, we determined the prognostic significance of L1CAM in cervical cancer and its association with vimentin expression on tumor cells, indicative of EMT.

**Methods:**

Formalin-fixed, paraffin-embedded primary tumor samples from 372 cervical cancer patients were collected for immunohistochemical analysis of L1CAM expression. In 109 FFPE specimens, the percentage of vimentin expressing tumor cells was determined by flow cytometry.

**Results:**

Positive L1CAM expression (≥10% of tumor cells) was associated with disease-free survival, validated using RNAseq TCGA data. L1CAM expression was independently associated with locoregional recurrence-free survival (hazard ratio 2.62, 95% CI 1.33 – 5.17, *P* = 0.006), and strongly associated with percentage of vimentin expressing tumor cells (*P* = 0.003). Expression of both L1CAM and vimentin indicated a subgroup with the highest risk of recurrence (hazard ratio 3.15, 95% CI 1.25 – 7.92, *P* = 0.015).

**Conclusion:**

L1CAM might be a promising new prognostic marker for locoregional recurrences in cervical cancer, and its association with vimentin expression suggests that L1CAM might affect tumor aggressiveness, possibly through EMT.

## INTRODUCTION

Cervical cancer is the fourth most common type of cancer among women worldwide [1]. Cervical cancer spreads in a progressive and predictable manner through regional lymphatics, suggesting that disease-recurrence is a result of insufficient primary treatment. For stage IB-IIA cervical cancer, the primary treatment is either primary radical surgery followed by adjuvant (chemo)radiotherapy, or primary chemoradiotherapy. Adjuvant radiotherapy after primary radical surgery is indicated when two out of three pathologic factors are present: vaso-invasion, tumor size ≥4cm, and tumor invasion ≥2/3 or ≥15mm (Sedlis criteria), but the risk of recurrences is only reduced by ~50% [2, 3]. Aside from currently used pathologic factors, molecular tumor markers might be helpful in predicting disease-recurrence, thus improving the selection of patients requiring adjuvant treatment.

A promising new marker is L1 cell adhesion molecule (L1CAM) [4]. L1CAM was first reported to be a strong prognostic factor for metastases in cutaneous malignant melanoma [5]. A recent meta-analysis reviewed 37 studies on the association between L1CAM expression and survival parameters [6]. L1CAM was shown to be a prognostic factor for overall survival in colorectal cancer, ovarian cancer, neuroendocrine tumors, GIST, cholangiocarcinoma, renal cell cancer, non-small cell lung cancer (NSCLC), hepatocellular cancer, endometrial cancer and for disease-free survival in neuroblastoma, ovarian cancer, neuroendocrine tumors, gallbladder cancer, hepatocellular cancer and endometrial cancer. The most informative cut-off value in these different tumor types was >10% of L1CAM positive tumor cells, as determined by immunohistochemistry [6].

One suggested mechanism through which L1CAM influences tumor progression and metastasis formation is epithelial-mesenchymal transition (EMT) [7]. During this process, epithelial cells loose cell to cell junctions and apico-basal polarity, resulting in a migratory and invasive mesenchymal-like phenotype [4]. Zecchini *et al*., describe a dual role for L1CAM, as L1CAM supported cell-cell adhesion and enhanced apoptosis in nontransformed ovarian epithelial cells, while it inhibited cell-cell adhesion and apoptosis and promoted malignancy-related properties, such as cell proliferation, invasion, and transendothelial migration in ovarian carcinoma cells [8]. In NSCLC, L1CAM expression was positively correlated with vimentin, beta-catenin, and slug expression, but inversely correlated with E-cadherin expression [9]. In cervical cancer, human papilloma virus (HPV) oncogene E6 has been shown to induce a mesenchymal phenotype in a chemoresistant cervical cancer cell line (SiHaCR), with elevated levels of survivin, snail, slug, twist and vimentin and reduced levels of E-cadherin [10]. In cetuximab/chemotherapy-treated NSCLC, vimentin expression was significantly associated with shorter progression-free survival [11]. Similarly, in oral squamous cell carcinoma, vimentin expression was associated with lymph node metastasis and poor overall survival [12].

The aim of the present study was to determine the protein expression of L1CAM and its association with clinical parameters and patient survival in a large cohort of cervical cancer patients, as well as in a TCGA validation cohort. Furthermore, we determined the association between L1CAM expression and percentage of vimentin expressing tumor cells, as a marker for EMT.

## RESULTS

### L1CAM expression in relation to patient characteristics

**Figure 1:**
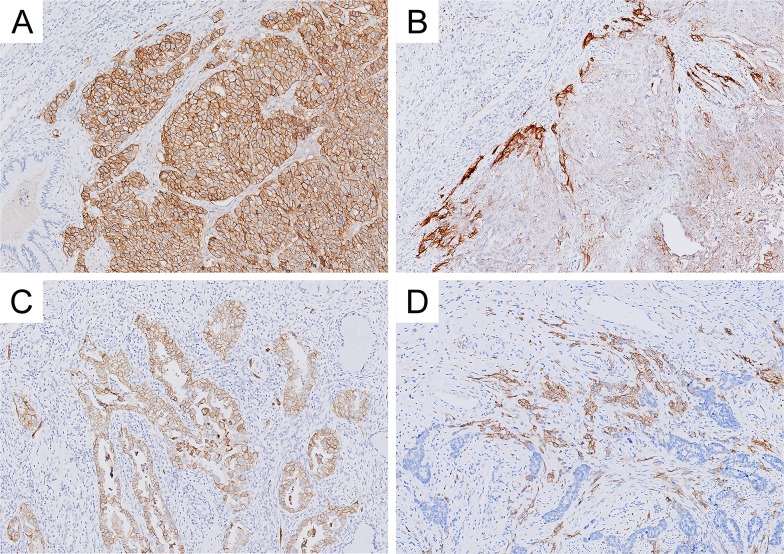
Representative examples of L1 cell adhesion molecule (L1CAM) expression in cervical carcinoma **(A)** diffuse staining pattern and **(B)** infiltration border positivity in squamous cell carcinoma, **(C)** diffuse staining pattern and **(D)** infiltration border positivity in adenocarcinoma.

Immunohistochemical staining of L1CAM in cervical cancer tissues was observed to be mainly membranous, accompanied in some cases by weak cytoplasmic staining. Samples with staining in less than 10% of the tumor cells, or samples with no L1CAM staining, were considered negative (N = 292, 79%). In 80 cases (21%), more than 10% of the tumor cells were L1CAM positive, of which 35 cases (9%) showed staining in more than 50% of the tumor. Of the L1CAM positive cases (≥10%), positive staining was observed exclusively at the infiltrating front in 42% of cases, while 34% showed a diffuse staining, including the infiltrating front. A minority of cases (5%) showed subclonal staining and 19% showed a heterogeneous staining pattern. Normal cervical epithelium in the proximity of cancerous tissue was negative for L1CAM expression. Representative examples of L1CAM staining are shown in Figure 1.

Clinicopathologic characteristics in relation to L1CAM expression are shown in Table 1. The median age at time of diagnosis was 43 years (range 24-87). The majority of patients underwent a radical hysterectomy with lymph node dissection (N = 347, 93%); a minority underwent an abdominal hysterectomy with or without adnex extirpation (N = 9, 3%) or radical trachelectomy with lymph node dissection (N = 16, 4%). Adjuvant therapy was administered to 187 patients (50%), of which 170 received radiotherapy and 17 received chemoradiotherapy. L1CAM expression (≥10%) was observed more frequently in HPV18 positive cases (38%), when compared to HPV16 positive cases (11%; Odds Ratio (OR) 4.8, 95% confidence interval (CI) 2.4 – 9.7, *P* <0.001). Furthermore, L1CAM expression (≥10%) was observed more frequently in adenocarcinoma (37%) and adenosquamous carcinoma (33%) than in squamous cell carcinoma (15%; squamous cell carcinoma vs. adeno(squamous) carcinoma: OR = 3.2, 95% CI 1.9 – 5.4, *P* <0.001). L1CAM expression was not associated with any of the other clinicopathologic characteristics.

**Table 1:** Clinicopathologic characteristics in relation to L1 cell adhesion molecule (L1CAM) expression

	Total	L1CAM <10%	L1CAM ≥10%	
	*N* ^a^ (%)	*N* (%)	*N* (%)	*P-value*
Age, yrs				
20-35	92 (25)	67 (73)	25 (27)	
36-45	118 (32)	93 (79)	25 (21)	
46-55	79 (21)	63 (80)	16 (20)	
56-100	83 (22)	69 (83)	14 (17)	0.409
HPV type				
Negative	32 (9)	21 (66)	11 (34)	
16	177 (48)	157 (89)	20 (11)	
18	58 (16)	36 (62)	22 (38)	
Other	45 (12)	32 (71)	13 (29)	**<0.001**
Unknown	60 (16)			
FIGO				
IA	4 (1)	3 (75)	1 (25)	
IB	307 (83)	241 (79)	66 (21)	
IIA	52 (14)	41 (79)	11 (21)	
IIB	8 (2)	7 (88)	1 (12)	
IIIA	1 (<1)	0 (0)	1 (100)	0.397
Histopathology				
SCC	266 (72)	225 (85)	41 (15)	
AS	12 (3)	8 (67)	4 (33)	
A	94 (25)	59 (63)	35 (37)	**<0.001**
Tumor size				
< 40 mm	228 (61)	182 (80)	46 (20)	
≥ 40 mm	121 (33)	92 (76)	29 (24)	0.412
Unknown	23 (6)			
Infiltration depth				
< 15 mm	224 (60)	173 (77)	51 (23)	
≥ 15 mm	136 (37)	110 (81)	26 (19)	0.413
Unknown	12 (3)			
Parametrial invasion				
Negative	316 (85)	247 (78)	69 (22)	
Positive	42 (11)	34 (81)	8 (19)	0.680
Unknown	14 (4)			
Vasoinvasion				
Negative	153 (41)	116 (76)	37 (24)	
Positive	197 (53)	159 (81)	38 (19)	0.268
Unknown	22 (6)			
Lymph node metastasis				
Negative	261 (70)	206 (79)	55 (21)	
Positive	109 (29)	85 (78)	24 (22)	0.840
Unknown	2 (1)			

^a^ Total number of cases = 372. *P*-value obtained with the Chi-square test.

HPV: human papillomavirus, FIGO: International Federation of Gynaecology and Obstetrics stage for cervical carcinoma, SCC: squamous cell carcinoma, AS: adenosquamous carcinoma, A: adenocarcinoma.

### L1CAM expression in relation to patient survival

Median follow-up time was 88 months (range 0–344) for all patients and 109 months (range 48–344) for the 216 patients alive at the time of data collection. Of the 120 patients that died during the follow-up period, 68 deaths could be attributed to cervical cancer. Thirty-six patients emigrated and were censored from the date of last follow-up. Of the 102 patients with disease recurrence, locoregional recurrences were observed in 45 cases, distant metastases in 48 cases and both locoregional and distant metastases in 9 cases.

**Figure 2:**
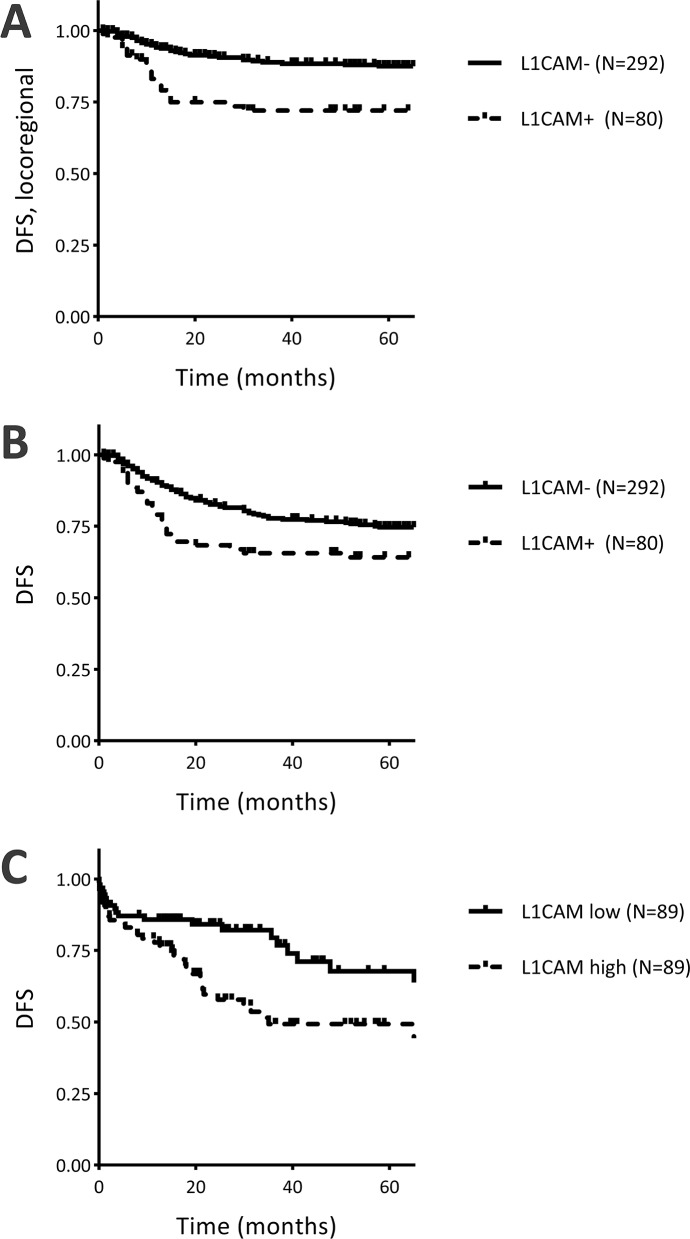
Survival curves for L1 cell adhesion molecule (L1CAM) expression and **(A)** locoregional recurrence-free survival in the research cohort; **(B)** disease-free survival in the research cohort; and **(C)** disease-free survival in the validation cohort (TCGA data). DFS = disease-free survival; L1CAM- = protein expression <10%; L1CAM+ = protein expression ≥10%; L1CAM low = mRNA expression below median; L1CAM high = mRNA expression above median.

Univariate Cox-regression analysis for L1CAM expression and disease-specific survival and disease-free survival is shown in Table 2. Expression of L1CAM was significantly associated with disease-free survival (Hazard Ratio (HR) 1.69, 95% CI 1.10 – 2.60, *P* = 0.017, Table 2, Figure 2B). Within the subgroup of patients that did not receive adjuvant radiotherapy, the association with disease-free survival was even stronger (*N* = 185, HR = 2.80, 95% CI 1.38 – 5.68, *P* = 0.004, Supplementary Figure 1A), while the association was no longer significant in the subgroup that received adjuvant radiotherapy (*N* = 187, HR = 1.29, 95% CI 0.74 – 2.25, *P* = 0.375, Supplementary Figure 1B). Subdivision of the recurrences into locoregional recurrence or distant metastasis showed that the association with disease-free survival was based on a strong association between L1CAM expression and locoregional recurrence-free survival (HR 2.86, 95% CI 1.66 – 4.93, *P* <0.001, Table 2, Figure 2A). After selection of patients with free resection margins, the association between L1CAM expression and locoregional recurrences was even stronger (HR 4.10, 95% CI 1.83 – 9.17, *P* = 0.001). Distant metastasis-free survival was not associated with L1CAM expression (HR 0.92, 95% CI 0.46 – 1.82, *P* = 0.808).

**Table 2:** Survival analyses for L1 cell adhesion molecule (L1CAM) expression

	Disease-specific survival				Disease-free survival				Locoregional recurrence-free survival			
	*N* events	HR	95% CI	*P*-value	*N* events	HR	95% CI	*P*-value	*N* events	HR	95% CI	*P*-value
L1CAM (*univariate)*												
<10%	49 (17%)	*Reference*			73 (25%)	*Reference*			32 (11%)	*Reference*		
≥10%	19 (24%)	1.61	0.95 – 2.74	0.077	22 (28)	**1.69**	**1.10 – 2.60**	**0.017**	22 (28%)	**2.86**	**1.66 – 4.93**	**<0.001**
L1CAM (*multivariate)*												
<10%		*Reference*				*Reference*				*Reference*		
≥10%		**1.99**	**1.08 – 3.66**	**0.028**		1.51	0.91 – 2.49	0.112		**2.62**	**1.33 – 5.17**	**0.006**
HPV type												
Negative										*Reference*		
16										0.60	0.21 – 1.74	0.346
18										1.02	0.34 – 3.03	0.971
Other										1.24	0.40 – 3.88	0.708
Tumour size												
< 40 mm		*Reference*				*Reference*				*Reference*		
≥ 40 mm		**2.00**	**1.13 – 3.54**	**0.017**		**2.04**	**1.29 – 3.22**	**0.002**		1.88	0.98 – 3.59	0.057
Infiltration depth												
< 15 mm		*Reference*				*Reference*						
≥ 15 mm		1.48	0.81 – 2.69	0.198		1.52	0.95 – 2.44	0.083				
Parametrial invasion												
Negative		*Reference*				*Reference*				*Reference*		
Positive		1.47	0.72 – 2.98	0.293		1.77	0.97 – 3.22	0.064		0.74	0.23 – 2.36	0.613
Vasoinvasion												
Negative		*Reference*				*Reference*						
Positive		1.10	0.56 – 2.18	0.778		1.10	0.65 – 1.86	0.719				
Lymph node metastasis												
Negative		*Reference*				*Reference*				*Reference*		
Positive		**3.09**	**1.58 – 6.06**	**0.001**		**1.98**	**1.16 – 3.38**	**0.012**		**1.75**	**0.84 – 3.65**	**0.133**

N = 372, L1CAM positive cases (≥10%) = 80, L1CAM negative cases (<10%) = 292.

Established prognostic factors were included in multivariate analyses if *P* <0.10 in univariate analysis. The following factors were considered: age, HPV type, histopathological diagnosis, tumour size, infiltration depth, parametrial invasion, vasoinvasion, lymph node metastasis.

^*^ Multivariate analysis for L1CAM expression using all established prognostic factors in the model, independent of significance in univariate analysis.

*N* events = number of events (%), HR = hazard ratio, 95% CI = 95% confidence interval, OS = overall survival, DSS = disease-specific survival, DFS = disease-free survival.

Multivariate Cox-regression analysis for L1CAM expression and established prognostic factors showed that L1CAM expression was independently associated with poor disease-specific survival (HR 1.99, 95% CI 1.08 – 3.66, *P* = 0.028, Table 2) and L1CAM expression was the only significant prognostic factor for locoregional recurrence (HR 2.62, 95% CI 1.33 – 5.17, *P* = 0.006, Table 2). Multivariate analysis using all prognostic factors, independent of significance in univariate analysis, showed that L1CAM expression was independently associated with disease-specific survival (HR 2.21, 95% CI 1.12 – 4.35, *P* = 0.022), disease-free survival (HR 1.80, 95% CI 1.01 – 3.18, *P* = 0.045) and locoregional recurrence-free survival (HR 2.48, 95% CI 1.21 – 5.09, *P* = 0.014).

**Figure 3:**
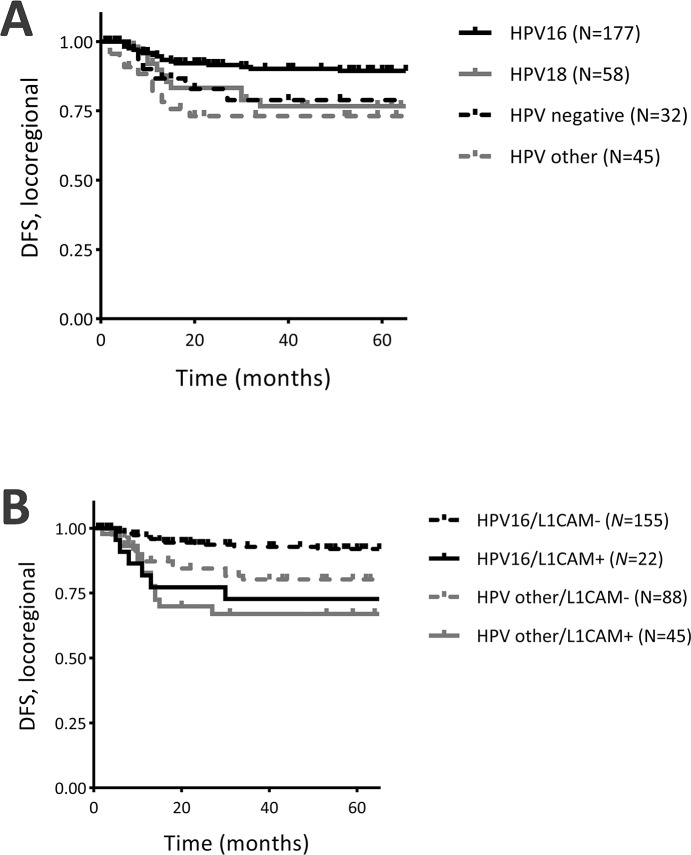
Survival curves for locoregional recurrence-free survival and **(A)** human papillomavirus type (HPV), **(B)** stratification for L1CAM and HPV16 / HPV other. DFS = disease-free survival; L1CAM- = L1CAM expression <10%; L1CAM+ = L1CAM expression ≥10%; HPV other = HPV18, other HPV types and HPV negative combined.

Univariate Cox-regression analysis for HPV type and locoregional recurrences showed that HPV16 positive cases had significantly less locoregional recurrences, when compared to cases with other HPV types (HR 0.35, 95% CI 0.16 – 0.73, *P* = 0.006, Figure 3A), while HPV18 positive cases (HR 0.74, 95% CI 0.32 – 1.70, *P* = 0.470) and HPV negative cases (HR 0.69, 95% CI 0.26 – 1.87, *P* = 0.470) showed a similar risk of locoregional recurrences, when compared to cases with other HPV types. As HPV type was also associated with L1CAM expression (Table 1), stratification for HPV type and L1CAM expression was performed to ascertain whether the association between L1CAM expression and locoregional recurrences was dependent on HPV type. Stratification for L1CAM expression and HPV16 or HPV other types (HPV18, other types, HPV negative) showed that HPV16+L1CAM- cases (reference) had the lowest risk of locoregional recurrences, when compared to HPV16+L1CAM+ cases (HR 5.27, 95% CI 2.04 – 13.59, *P* = 0.001), HPVother+L1CAM- cases (HR 2.71, 95% CI 1.24 – 5.90, *P* = 0.012) and HPVother+L1CAM+ cases (HR 4.89, 95% CI 2.19 – 10.93, *P* <0.001; Figure 3B). Histopathological type was not associated with locoregional recurrence-free survival, or any of the other survival parameters.

### L1CAM expression in relation to percentage of vimentin expressing tumor cells

**Figure 4:**
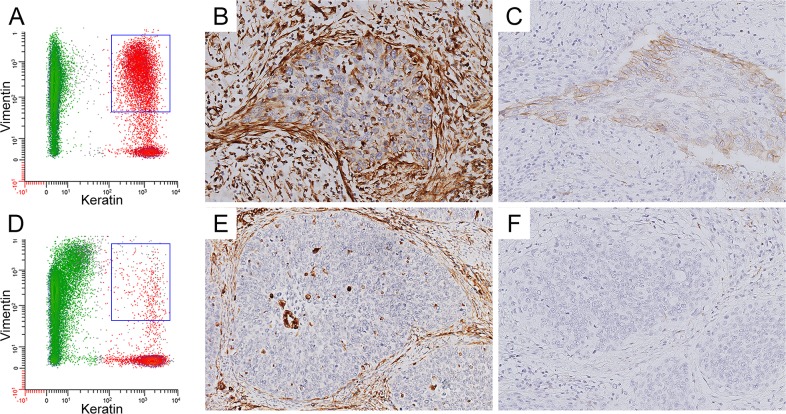
**(A+D)** Representative FACS analysis of tumor samples with high (A) and low (D) fraction of vimentin positive tumor cells. The keratin negative fraction is shown in green, the keratin positive fraction (i.e. tumor fraction) is shown in red. The keratin/vimentin double-positive tumor fraction is indicated with a blue square. **(B+E)** Immunohistochemical staining of high (B) and low (E) vimentin expression. **(C+F)** Immunohistochemical staining of high (C) and low (F) L1CAM expression.

As L1CAM expression has been related to EMT [9, 13–16], we assessed the association between L1CAM expression and the percentage of vimentin expressing (keratin positive) tumor cells, determined by flow cytometry. A subset of 109 cases was analyzed, with a median of 13.6% of tumor cells expressing vimentin, range 2.0 – 95.3%. Representative examples of a tumor sample showing double positive keratin/vimentin tumor cell subpopulation and a tumor sample without vimentin co-expression, with corresponding L1CAM and vimentin staining, are shown in Figure 4. Clinicopathologic characteristics in relation to the percentage of vimentin expressing tumor cells are shown in Supplementary Table 1. Percentage of vimentin expressing tumor cells was strongly associated with L1CAM expression (*P* = 0.003, One-way ANOVA). Cases were subdivided into two groups, based on the 75^th^ percentile (26.1%) of vimentin expressing tumor cells. For L1CAM <10% cases, 19.5% showed high vimentin expression, compared to 45.5% of the L1CAM ≥10% cases. High vimentin expression was also observed more frequently in HPV18 positive cases (31.3%), HPV negative cases (50.0%) and HPV other cases (45.0%), when compared to HPV16 positive cases (14.1%; *P* = 0.010). Furthermore, high vimentin expression was observed more frequently in higher FIGO stages (46.2% in FIGO stage IIA and higher, versus 18.1% in FIGO stage IB and lower; *P* = 0.004). Percentage of vimentin expressing tumor cells was not associated with histopathological diagnosis, or any of the other clinicopathologic parameters.

For survival analysis, patients were stratified according to L1CAM expression and percentage of vimentin expressing tumor cells. The number of events in the subgroups was too low for survival analysis for locoregional recurrence-free survival. Therefore, disease-free survival analysis was performed. L1CAM-/vimentin- cases (reference) had the lowest risk of recurrence, when compared to L1CAM-/vimentin+ cases (HR 1.47, 95% CI 0.59 – 3.67, *P* = 0.415), L1CAM+/vimentin- cases (HR 1.79, 95% CI 0.67 – 4.81, *P* = 0.247) and L1CAM+/vimentin+ cases (HR 3.15, 95% CI 1.25 – 7.92, *P* = 0.015).

### Validation cohort

In order to validate these findings in an independent cohort, RNAseq data were used from 178 cervical cancer cases, published by the Cancer Genome Atlas Research Network [17, 18]. L1CAM expression was divided into two groups based on median L1CAM mRNA expression (309.354, range 1 – 140.750.861). Clinicopathologic characteristics in relation to L1CAM expression are shown in Supplementary Table 2. The median age at time of diagnosis was 46 years (range 20-88). L1CAM mRNA expression was significantly associated with five-year disease-free survival (HR 1.86, 95% CI 1.08 – 3.19, *P* = 0.025, Figure 2C). Furthermore, L1CAM mRNA expression was strongly correlated with the EMT score (*P* <0.001), as determined by the level of *fibronectin*,*N-cadherin*, *collagen-IV* and *PAI-1* upregulation and *claudin-7*,*E-cadherin* and *β-catenin* downregulation. The EMT score has been described and validated elsewhere [17, 19].

## DISCUSSION

We investigated the association between L1CAM expression and survival, as well as the association between L1CAM expression and percentage of vimentin expressing tumor cells. Our data show that L1CAM expression (≥10%) was a strong independent predictor of worse locoregional recurrence-free survival in 372 cervical cancer patients (HR 2.62, 95% CI 1.33 – 5.17, *P* = 0.006). L1CAM expression was strongly associated with percentage of vimentin expressing tumor cells (*P* = 0.003), and expression of both L1CAM and vimentin indicated a subgroup with the highest change of recurrence (HR 3.15, 95% CI 1.25 – 7.92, *P* = 0.015).

This is the first study reporting on the expression of L1CAM in cervical cancer specimens. L1CAM expression (≥10%) was observed in 21% of cases. Studies on other gynecological cancer types reported 43% positivity in ovarian cancer (membrane+) [8], 7 – 44% positivity in endometrial cancer (≥10%) [13, 20–25], and 16% positivity in vulvar cancer (≥10%) [26]. Expression of L1CAM has been reported as an independent prognostic factor for disease-free survival in ovarian cancer [27], neuroendocrine tumors [28], gallbladder cancer [29], hepatocellular cancer [30] and endometrial cancer [20]. However, the site of recurrence (locoregional versus distant) is not specified in these studies. So far, reliable prognostic markers that can identify patients with high risk of locoregional recurrence are rare. In a cohort of 95 stage 1B cervical cancer patients undergoing radical hysterectomy and pelvic lymphadenectomy, only tumor differentiation was an independent predictive factor for recurrent disease, but sole use of this feature as a criterion for adjuvant therapy would have resulted in overtreatment of low-risk patients, as almost half of patients had poorly differentiated tumors [31]. In our cohort, L1CAM expression (≥10%) was observed in 80 cases, of which 22 cases (28%) developed a locoregional recurrence, thus preselecting patients with high risk of recurrent disease, independent of the status of the resection margins. The prognostic value of L1CAM was stronger than currently established prognostic factors, such as status of resection margins and tumor size.

L1CAM might be a promising new target for antibody-based therapy in human cancers, as reviewed by Altevogt *et al* [32]. In mouse models for ovarian cancer, pancreatic cancer and cholangiocarcinoma, L1CAM antibodies significantly prolonged survival, reduced ascites formation and reduced tumor burden [33–36]. In our cohort, L1CAM expression was strongly associated with locoregional recurrence-free survival in patients that did not receive adjuvant radiotherapy, while the association was no longer present in the subgroup of patients that received adjuvant radiotherapy, suggesting that L1CAM positive tumors are susceptible to radiotherapy. Therefore, these results indicate that L1CAM expression might also be used to select cervical cancer patients with a high risk of locoregional recurrences, that might benefit from adjuvant radiotherapy.

As L1CAM is thought to play a role in EMT [7, 9, 13–16], we investigated the association between L1CAM expression and percentage of vimentin expressing tumor cells, assessed by flow cytometry. L1CAM expression was strongly associated with vimentin expression. Cell line studies in human pancreatic ductal adenocarcinoma, endometrial, breast and lung cancer cells showed that treatment with TGF-β1 initiated transcription factor slug, not only resulting in upregulated L1CAM/vimentin and downregulated E-cadherin, but also in enhanced cell invasion [9, 13–16]. Furthermore, Shtutman *et al*. showed that L1CAM expression leads to the disruption of adherens junctions and increases B-catenin transcriptional activity in MCF7 breast carcinoma cells [37]. In cervical cancer cell lines, such as HeLa, overexpression of L1CAM was significantly associated with differentiation. RNAi-mediated knockdown of LICAM decreased the proliferation, migration and invasion of cervical cancer cells while over-expression of L1CAM increased proliferation, migration and invasion [38].

In cervical cancer, several mechanisms are potentially involved in EMT induction, including epigenetic factors, low dose radiation, HPV oncogenes E6 and E7 and TGF-β expression, through transcription factors twist, ZEB1, snail/slug, and several matrix metalloproteinases [39]. Tumor-associated macrophages are suggested to play an important role in the induction of EMT [40, 41]. Our cervical cancer cohort has been extensively studied, and many immune parameters are known, including the presence of infiltrating myeloid cells and their relationship to other tumor-infiltrating immune cells, tumor characteristics and patient survival [42–44]. L1CAM expression was associated with the presence of CD14+CD33+CD163+ macrophages (*P* = 0.040, N = 52, Fisher's Exact Test) and the expression of plasminogen activator inhibitor 1 (PAI-1; *P* = 0.010, N = 55, Fisher's Exact Test). These data support published cell line studies that show that L1CAM upregulation is initiated by CD33+ macrophages, through TGF-β1 in a slug-dependent fashion [45, 46]. Understanding EMT in cervical cancer is of prime importance, as EMT leads to chemoresistance and radioresistance [47, 48]. Based on the link found between macrophage infiltration and TGF-beta activation in the present cohort, further functional analyses are warranted and much needed to investigate the possible role of L1CAM in EMT in cervical cancer.

In our cohort, L1CAM expression was higher in adeno(squamous) carcinoma (37%), when compared to squamous cell carcinoma (15%). This has also been observed in other tumor types, such as esophageal cancer [49]. Furthermore, L1CAM expression was associated with HPV type, with lower expression in HPV16 positive tumors (11%) than in HPV18 positive tumors (38%). In vulvar cancer, L1CAM expression was not associated with HPV [26], but little is known about the association between L1CAM and HPV infection in other cancer types. However, HPV E6/E7-transfected cervical cells showed upregulated vimentin expression and downregulated E-cadherin protein expression, suggesting a possible role for HPV in the development of EMT in cervical cancer [50]. In MDCK cells, stable expression of HPV16-E6 or E7 induced increased expression of transcription factors slug, Twist, ZEB1 and ZEB2, accompanied with a switch from epithelial to mesenchymal markers, and a migratory and invasive phenotype [51]. A chemoresistant cervical cancer cell line (SiHaCR) showed increased levels of HPV E6 and E7 transcripts and a mesenchymal phenotype, with upregulated snail/slug/twist/vimentin and downregulated E-cadherin. Specific silencing of E6, but not E7, resulted in a more epithelial phenotype and reduced migration and invasion potential [10]. These results suggest, that the HPV E6 oncogene might play an important role in the development of EMT in cervical cancer. Further research is needed to investigate the relation between HPV and L1CAM expression.

In conclusion, L1CAM might be a promising new prognostic marker for locoregional recurrences in cervical cancer, independent of currently established prognostic markers. Furthermore, L1CAM expression was strongly associated with percentage of vimentin expressing tumor cells and expression of both L1CAM and vimentin indicated a subgroup with the highest change of recurrence, suggesting that L1CAM ascertains tumor aggressiveness, possibly through EMT.

## MATERIALS AND METHODS

### Subjects and follow-up data

Formalin-fixed, paraffin-embedded primary tumor samples from 372 cervical cancer patients, who underwent surgical treatment between 1985 and 2011, were retrospectively retrieved from the archives of the Department of Pathology, Leiden University Medical Center, Leiden, the Netherlands. Patients were treated according to local protocol. Patients that had received radiotherapy and/or chemotherapy prior to surgery were excluded. Clinical and follow-up data were collected through the hospital-wide oncology database. Follow-up data in this database are recorded prospectively in a de-identified manner, and are updated directly from patient medical records. Follow-up was recorded from the date of primary surgery to September 2016. Follow-up time was considered as the time in months between primary surgery and: 1) death by cervical cancer, with patients who died of a cause unrelated to cervical cancer considered as censored observations at the date of death (disease-specific survival); and 2) locoregional recurrence or distant metastasis, which ever occurred first, when both occurred within 30 days, both are stated, death is considered as a censored observation (disease-free survival).

HPV detection was performed by PCR using CP-I/II, GP5þ/6þ and MY09/MY11 consensus primers. Samples that were found to be positive for HPV were subsequently sequenced to determine the HPV genotype [52]. For a part of the cohort HPV DNA was amplified using the SPF10 primer set, and HPV DNA detection and broad spectrum HPV genotyping were performed using INNO-LiPA HPV genotyping Extra line probe assay (Innogenetics, Ghent, Belgium) [53]. Histopathological diagnosis was confirmed by review of hematoxylin-eosin slides and Periodic Acid-Schiff (PAS) reagent / Alcian Blue (AB) staining by a senior pathologist. Tissue samples were used according to the guidelines of the Ethical Committee of the Leiden University Medical Center.

### Immunohistochemistry

Four-μm paraffin-embedded tissue sections were deparaffinized and rehydrated. Endogenous peroxidase was blocked with 0.3% H_2_O_2_ for 20 minutes. Antigen retrieval was performed in Tris-EDTA (pH 9.0). Slides were incubated overnight at room temperature with mouse monoclonal anti-L1CAM (1:500, IgG1, clone 14.10, BioLegend), and subsequently for 30 min with PowerVision-Poly/HRP (Immunologic, Duiven, the Netherlands). Immunoreactions were visualized using 0.5% 3.30-diamino-benzidine-tetrahydrochloryde (DAB) and 0.002% H2O2 in Tris-HCl, and counterstained with hematoxylin. The percentage of L1CAM positive tumor cells was scored independently by two authors, who were blinded to clinical data. A cut-off value of 10% was used for further analysis, as this has been shown to be the most informative cut-off. Nerve cells were used as an internal positive control.

### The Cancer Genome Atlas (TCGA) RNAseq data as a validation cohort

Level 3 RSEM normalized RNA data, profiled using the Illumina HiSeq RNAseq v2, were retrieved from the TCGA data portal. Results of the TCGA RNAseq analysis have been described in detail by the Cancer Genome Atlas Research Network [17]. For our analysis, data on 178 cervical cancer patients were used, including clinical data, L1CAM mRNA expression and an EMT score. The EMT score was computed as described in the original article [17, 19]. Briefly, the EMT score was the value resulting from the difference between the average expression of mesenchymal genes (*fibronectin*, *N-cadherin*, *collagen-VI* and *PAI-1*) and the average expression of epithelial genes (*claudin-7*, *E-cadherin* and *β-catenin*).

### Flow cytometric analysis

A subset of 109 FFPE cervical cancer specimens were randomly collected from the cohort stained for L1CAM by IHC, based on tumor material availability, by an investigator blinded to L1CAM expression. Of these, 22 cases (20%) were positive for L1CAM expression based on the immunohistochemical staining described above. Flow cytometry was performed as previously described [18]. In short, cell suspensions were prepared from 2-3 dewaxed 60 μm FFPE material sections. One million cells were incubated with 100 μl of MAb mixture directed against keratin (clone MNF116, 2 μg/mL, DAKOCytomation, Glostrup, Denmark and clone AE1/AE3, 5 μg/mL, Chemicon International Inc, Temecula, CA, USA), and vimentin (clone V9-2b, dilution 1:5 culture supernatant (Antibodies for Research Applications, Gouda, Netherlands) overnight at 4°C. Cells were washed and incubated with 100 μl of premixed subclass-specific FITC or APC-labelled secondary reagents (Southern Biotechnology Associates, Birmingham, AL, USA). DNA was labelled with PI and RNase (Sigma-Aldrich) treated (analyzed using a FACSCalibur flow cytometer, BD Biosciences, San Jose, CA) or DAPI (analyzed using an LSRII flow cytometer BD Biosciences). A minimum of 30,000 single cell events were collected for each sample. A data file contained all events, included aggregates and debris, and data were analyzed using the WinList 8.0 and Mod-Fit 4.1 software packages (Verity Software House, Inc, Topsham, ME, USA). The fraction of keratin-positive/vimenitin-negative (K+) and keratin-positive/vimentin-positive (V+) cells was calculated after gating on single cells in the DNA-Area versus DNA-Width (doublet discrimination module) dot plot.

### Statistical analyses

To assess whether L1CAM expression was associated with clinicopathologic characteristics, the chi-squared (χ^2^) test was used. Univariate Cox-regression analysis was performed to assess the association with disease-free survival / disease specific survival. Survival curves were estimated by the Kaplan-Meier method. Multivariate Cox-regression analysis was performed to determine whether L1CAM expression was independently associated with survival, for which age, HPV type, histopathological diagnosis, tumor size, infiltration depth, parametrial invasion, vaso-invasion and lymph node metastasis were considered as covariates and included in multivariate analyses if *P* <0.10 in univariate analysis. To determine the association between L1CAM expression and percentage of vimentin expressing tumor cells, one-way ANOVA was used, as data were not normally distributed. Vimentin expression was also subdivided into two groups, based on the 75^th^ percentile (26.1%) of vimentin expressing tumor cells. The association between vimentin expression and clinicopathologic characteristics was assessed using the chi-squared (χ^2^) test. Significance tests were two-sided and statistical significance was assumed when *P* <0.05, corresponding to 95% confidence intervals (CI). Statistical analyses were performed using IBM SPSS Statistics 23. This study is reported according to Reporting recommendations for tumor MARKer prognostic studies (REMARK) [54].

## SUPPLEMENTARY MATERIALS FIGURE AND TABLES



## Abbreviations

95% CI: 95% confidence interval
AB: Alcian Blue
EMT: epithelial mesenchymal transition
FFPE: formalin-fixed paraffin-embedded
HPV: human papillomavirus
HR: hazard ratio
L1CAM: L1 cell adhesion molecule
NSCLC: non-small cell lung cancer
PAI1: plasminogen activator inhibitor 1
PAS: Periodic Acid-Schiff reagent
TCGA: The Cancer Genome Atlas
TGF-β1: transforming growth factor beta-1
