# Supplementary material for: L1 cell adhesion molecule (L1CAM) is a strong predictor for locoregional recurrences in cervical cancer
Source: Oncotarget. 2017 Sep 18;8(50):87568–81. doi: 10.18632/oncotarget.20976 (PMC5675654; doi:10.18632/oncotarget.20976)
Supplement: Supplementary file 1 [file oncotarget-08-87568-s001.pdf]

L1 cell adhesion molecule (L1CAM) is a strong predictor for locoregional recurrences in cervical cancer

SUPPLEMENTARY MATERIALS

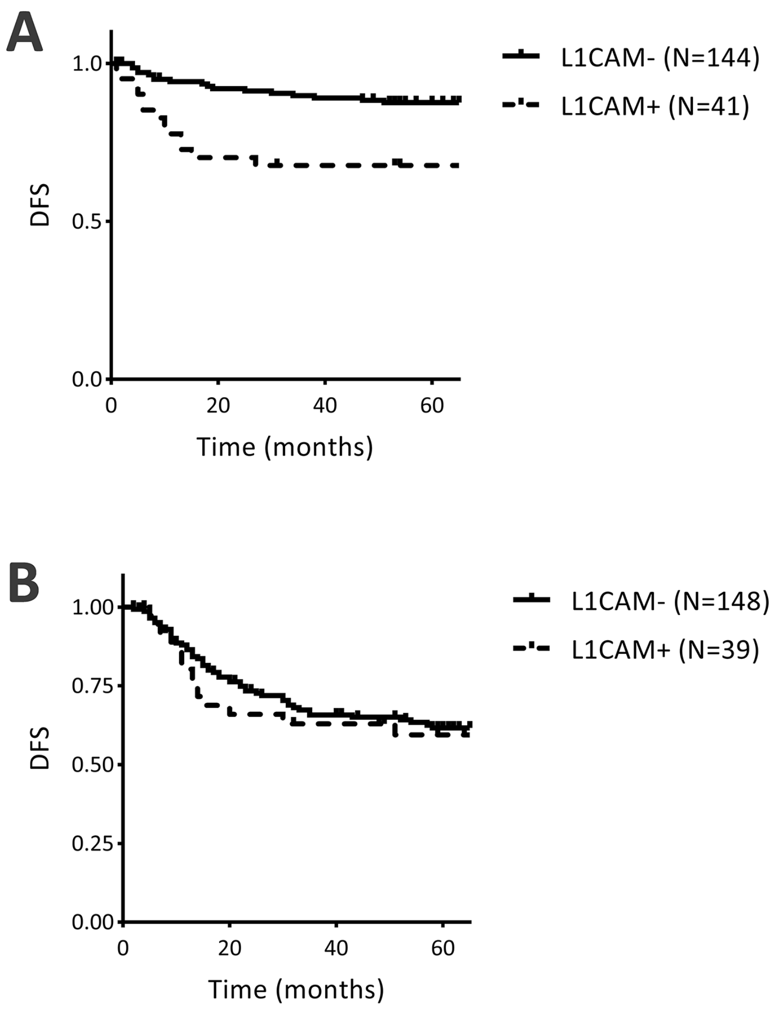

Supplementary Figure 1: Survival curves for disease-free survival and L1CAM expression in subgroups of patients without **(A)** and with **(B)** adjuvant therapy. DFS = disease-free survival, L1CAM- = protein expression <10%; L1CAM+ = protein expression ≥10%.

Supplementary Table 1: Clinicopathologic characteristics in relation to vimentin expression

	Total	Vimentin low*	Vimentin high*	
	N (%)**	*N* (%)	*N* (%)	*P-value*
L1CAM				
<10%	87 (80)	70 (81)	17 (19)	
≥10%	22 (20)	12 (55)	10 (45)	**0.012**
Age, yrs				
20-35	24 (22)	17 (71)	7 (29)	
36-45	33 (30)	25 (76)	8 (24)	
46-55	26 (24)	17 (65)	9 (35)	
56-100	26 (24)	23 (89)	3 (11)	0.256
HPV type				
Negative	8 (7)	4 (50)	4 (50)	
16	64 (59)	55 (86)	9 (14)	
18	16 (15)	11 (69)	5 (31)	
Other	20 (18)	11 (55)	9 (45)	**0.010**
Unknown	1 (1)			
FIGO				
IB	83 (76)	68 (82)	15 (18)	
IIA	21 (19)	12 (57)	9 (43)	
IIB	4 (4)	2 (50)	2 (50)	
IIIA	1 (1)	0	1	**0.018**
Histopathology				
SCC	89 (82)	67 (75)	22 (25)	
A	20 (18)	15 (75)	5 (25)	0.979
Tumor size				
< 40 mm	44 (40)	34 (77)	10 (23)	
≥ 40 mm	59 (54)	43 (73)	16 (27)	0.612
Unknown	6 (6)			
Infiltration depth				
< 15 mm	54 (50)	39 (72)	15 (28)	
≥ 15 mm	50 (46)	41 (82)	9 (18)	0.237
Unknown	5 (4)			
Parametrial invasion				
Negative	92 (84)	70 (76)	22 (24)	
Positive	16 (15)	12 (75)	4 (25)	0.925
Unknown	1 (1)			
Vasoinvasion				
Negative	45 (41)	33 (73)	12 (27)	
Positive	57 (52)	45 (79)	12 (21)	0.507
Unknown	7 (7)			
Lymph node metastasis				
Negative	70 (64)	53 (76)	17 (24)	
Positive	39 (36)	29 (74)	10 (26)	0.875

* Vimentin expression was assessed by flow cytometry analysis. Cases were subdivided into two groups, based on the 75th percentile of vimentin expressing tumor cells.
** Total number of cases = 109. *P*-value obtained with the Chi-square test.
HPV: human papillomavirus, FIGO: International Federation of Gynaecology and Obstetrics stage for cervical carcinoma, SCC: squamous cell carcinoma, A: adenocarcinoma.

Supplementary Table 2: Clinicopathologic characteristics of the TCGA validation cohort in relation to L1 cell adhesion molecule (L1CAM) expression

	Total	L1CAM low	L1CAM high	
	N [a] (%)	*N* (%)	*N* (%)	*P-value*
Age, yrs				
20-35	34 (19)	15 (44)	19 (56)	
36-45	52 (29)	24 (46)	28 (54)	
46-55	49 (28)	23 (47)	26 (53)	
56-100	43 (24)	27 (63)	16 (37)	0.287
HPV type				
Negative	9 (5)	6 (67)	3 (33)	
16	103 (58)	54 (52)	49 (48)	
18	27 (15)	13 (48)	14 (52)	
Other	39 (22)	16 (41)	23 (59)	0.469
Unknown				
FIGO				
I	109 (61)	53 (49)	56 (51)	
II	32 (18)	15 (47)	17 (53)	
III-IV	34 (19)	18 (53)	16 (47)	
missing	3 (2)	3	0	0.344
Histopathology				
SCC	144 (81)	16 (52)	15 (48)	
AS	3 (2)	2 (67)	1 (33)	
A	31 (17)	71 (49)	73 (51)	0.821
Lymph node metastasis				
Negative	88 (49)	48 (55)	40 (45)	0.336
Positive	33 (19)	17 (52)	16 (48)	
Unknown	57 (32)	24 (42)	33 (58)	

[a] Total number of cases = 178. L1CAM expression is divided into two groups based on median mRNA expression. *P*-value obtained with the Chi-square test.
HPV: human papillomavirus, FIGO: International Federation of Gynaecology and Obstetrics stage for cervical carcinoma, SCC: squamous cell carcinoma, AS: adenosquamous carcinoma, A: adenocarcinoma.
